# Description of the Three Complete Mitochondrial Genomes of *Sitta* (*S. himalayensis*, *S. nagaensis,* and *S. yunnanensis*) and Phylogenetic Relationship (Aves: Sittidae)

**DOI:** 10.3390/genes14030589

**Published:** 2023-02-26

**Authors:** Qingmiao Yuan, Qiang Guo, Jing Cao, Xu Luo, Yubao Duan

**Affiliations:** 1 Key Laboratory for Conserving Wildlife with Small Populations in Yunnan, Southwest Forestry University, Kunming 650224, China; 2 Department of Biodiversity Conservation, Southwest Forestry University, Kunming 650224, China; 3 Administration of Zixi Mountain Provincial Nature Reserve, Chuxiong 675000, China

**Keywords:** *Sitta himalayensis*, *Sitta nagaensis*, *Sitta yunnanensis*, mitochondrial genome, phylogeny

## Abstract

Nuthatches (genus *Sitta*; family Sittidae) are a passerine genus with a predominantly Nearctic and Eurasian distribution. To understand the phylogenetic position of *Sitta* and phylogenetic relations within this genus, we sequenced the complete mitochondrial genomes of three *Sitta* species (*S. himalayensis*, *S. nagaensis,* and *S. yunnanensis*), which were 16,822–16,830 bp in length and consisted of 37 genes and a control region. This study recovered the same gene arrangement found in the mitogenomes of *Gallus gallus*, which is considered the typical ancestral avian gene order. All tRNAs were predicted to form the typical cloverleaf secondary structures. Bayesian inference and maximum likelihood phylogenetic analyses of sequences of 18 species obtained a well-supported topology. The family Sittidae is the sister group of Troglodytidae, and the genus *Sitta* can be divided into three major clades. We demonstrated the phylogenetic relationships within the genus *Sitta* (*S. carolinensis* + ((*S. villosa* + *S. yunnanensis*) + (*S. himalayensis* + (*S. europaea* + *S. nagaensis*)))).

## 1. Introduction

The avian mitochondrial genome is characterized by a small size, a relatively fast rate of evolution, and strict maternal inheritance. Avian mitogenomes are easy to extract and amplify, which makes them an ideal marker for the evolutionary analyses at the molecular level [1,2,3]. The avian mitogenome is a double-stranded circular molecule composed of a light strand (L-strand) and a heavy strand (H-strand) ranging from 16.3–23.1 kb in length [4] and consisting of 1 or 2 control region(s), 13 protein-coding genes (PCGs), 22 transfer RNA genes (tRNAs) and 2 ribosomal RNA genes (rRNAs) [5,6,7]. The mitogenome plays an important role in revealing the phylogeny of birds [8].

The genus *Sitta*, which belongs to the subfamily Sittinae (Passeriformes, Sittidae), includes 29 species of genus *Sitta* known around the world, 11 of which are distributed in China [9]. Although many molecular data have been published, the phylogenetic relationship of the family Sittidae remains controversial. Ericson and Johansson placed the family Sittidae within Sylvioidea [10]. In recent years, it has also been proposed that Sittidae belongs to Certhioidea [11,12]. Johansson et al. supported that Sittidae was sister to Polioptilidae and Troglodytidae [11], while Treplin et al. proposed that Sittidae was sister to Certhiidae and Troglodytidae [13]. Subsequent research results showed that Sittidae and Troglodytidae were closely related [14]. These controversies are mainly generated by limited mitochondrial genome data. Therefore, more molecular data are necessary to reconstruct a precise phylogeny [15].

Currently, complete mitogenome sequences from the genus *Sitta* are very scarce. In order to better understand the relationships among the species of *Sitta*, we sequenced three mitogenomes of the genus *Sitta* and compared them with related species in terms of mitochondrial structure and gene rearrangement. In this study, we report the properties of these three *Sitta* species and infer their phylogenetic relationships with available mitogenome sequences using all PCGs.

## 2. Materials and Methods

### 2.1. Samples and DNA Extraction

A frozen sample of *Sitta himalayensis* was provided by the Gaoligong Mountain Nature Park of Yunnan Province, China. Frozen samples of *Sitta nagaensis* and *Sitta yunnanensis* were provided by the Zixi Mountain Provincial Nature Reserve of Yunnan Province, China. Voucher samples of three *Sitta* species were deposited in the Department of Biodiversity Conservation, Southwest Forestry University, Kunming. Samples used in this study were preserved in ethanol absolute and stored at −20 °C. The total genomic DNA was extracted from blood using the TIANamp Genomic DNA Kit (DP304, TIANGEN, Beijing, China) according to the manufacturer’s protocol.

### 2.2. Genome Sequencing, Assembly, and Annotation

As described in previous studies, the mitogenomes were amplified and sequenced [16,17,18]. All products in this study were sequenced by Shanghai Personal Biotechnology Co., Ltd. (Shanghai, China). The complete mitogenomes of *S. himalayensis*, *S. nagaensis,* and *S. yunnanensis* were deposited in GenBank (Accession Nos.: MK343426, MK343427, and MN052793). Sequences were checked and assembled with the SeqMan program of the DNASTAR software [16]. Two rRNAs and all PCGs were identified by BLAST searches in NCBI (available online: http://www.ncbi.nlm.nih.gov) (accessed on 10 July 2022) and then confirmed via alignment with homologous genes from other published *Sitta* mitogenomes. The mitogenomic map was depicted with OGDRAW (https://chlorobox.mpimp-golm.mpg.de/OGDraw.html) (accessed on 10 July 2022) [19]. Then, 22 tRNAs were identified using tRNAscan-SE 2.0 and ARWEN (online version) [20,21]. MEGA 7.0 was used to calculate the nucleotide composition and relative synonymous codon usage (RSCU) for PCG analysis [22]. The formulas AT-skew = (A − T)/(A + T) and GC-skew = (G − C)/(G + C) were used for composition skew analysis [23]. The Tandem Repeats Finder program (available online: http://tandem.bu.edu/trf/trf.advanced.submit.html) (accessed on 10 July 2022) was used to analyze the tandem repeats of the putative control region [24]. Moreover, the genome organization, base composition, intergenic spacers, overlapping regions, codon usage, PCGs, tRNAs, rRNAs, and control region of the mitochondrial genomes of three *Sitta* species were compared.

### 2.3. Phylogenetic Analyses

The sequences of 15 published mitochondrial genomes were obtained from NCBI. These sequences—along with three new mitogenome sequences obtained in this study—were used to reconstruct the phylogenetic relationships within the genus *Sitta* using *Regulus regulus* (Accession No. NC_029837) as an outgroup [25,26]. In addition, an unpublished mitogenome sequence of *Cyanoptila cyanomelana* available in GenBank (HQ896033) was here named *Cyornis hainanus* or *C. rubeculoides* following clarification of its identity by Sangster and Luksenburg [8]. The sequence information is listed in Table 1.

The mitogenome sequences of the 13 PCGs were aligned using Clustal X in MEGA v.7.0 with the default parameters [34]. The length of the final alignment was 11,380 nucleotides. The substituted saturation of nucleotide sequences was analyzed with DAMBE 5.2.63 [35]. If Iss was significantly lower than Iss.c (*p* < 0.05), all of the PCG nucleotide sequences entered the next step. The Bayesian information criterion (BIC) in jModelTest v.0.1.1 [36] was used to determine the optimal nucleotide substitution model, which was GTR+G+I. Bayesian inference (BI) and maximum likelihood (ML) analyses were performed using MrBayes v.3.2.1 [37,38] and RAxMLGUI v.1.5b3 [39], respectively. BI analyses initiated from a random tree with four Markov chains running simultaneously for 200,000 generations and sampling every 100 generations and discarding the first 25% as burn-in. The average standard deviation of split frequencies was set below 0.01 to ensure that stationarity was reached [40]. The confidence values of the BI tree were shown as Bayesian posterior probabilities. In the ML analyses, a total of 1000 replicates were performed with the GTR+GAMMA substitution model. Finally, FigTree v.1.2.2 was used to visualize the phylogenetic trees [41].

## 3. Results

### 3.1. Genome Organization

**Figure 1:**
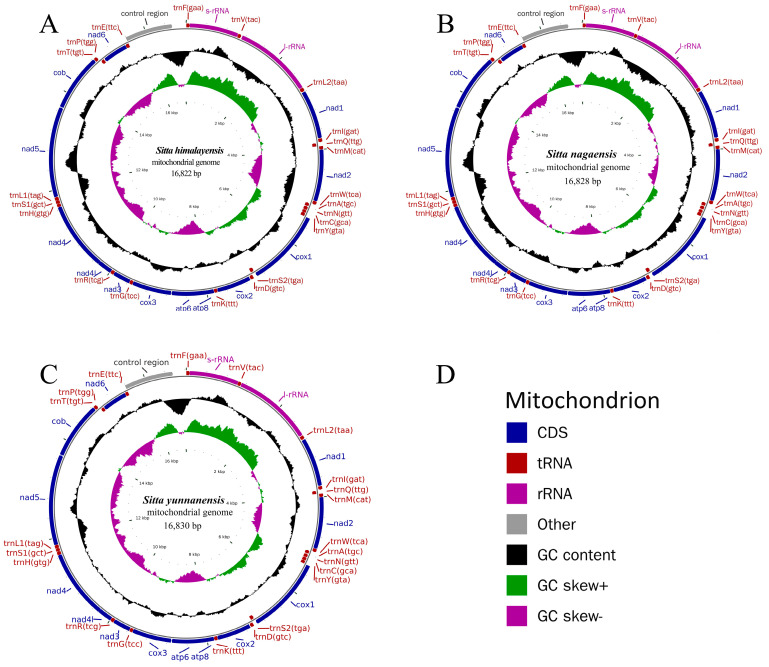
Characteristics of mitochondrial genomes of the three newly sequenced *Sitta* species. Gene names are annotated using standard abbreviations; single letters indicate corresponding amino acids based on the IUPAC-IUB abbreviation. (**A**) *S. himalayensis* mitochondrial genome; (**B**) *S. nagaensis* mitochondrial genome; (**C**) *S. yunnanensis* mitochondrial genome. The legend is depicted in (**D**).

**Figure 2:**
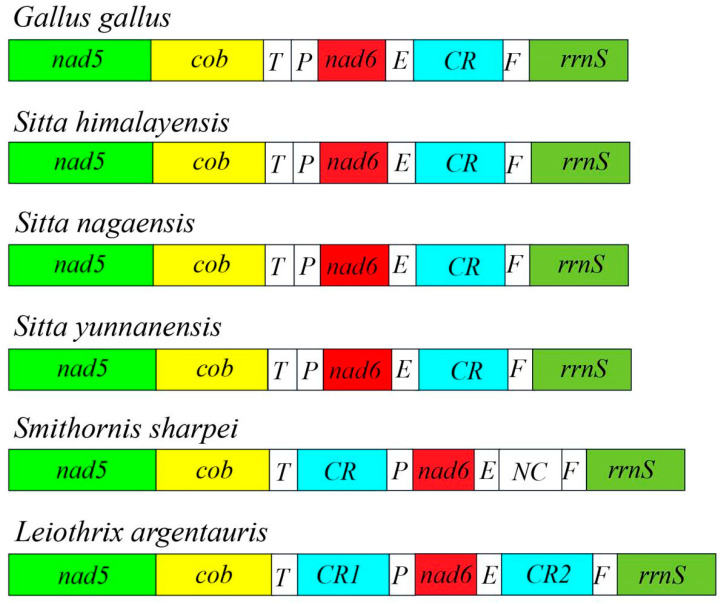
Mitochondrial gene order from nad5 to *12S rRNA* as in three *Sitta* species.

The complete mitogenomes of the three newly sequenced *Sitta* species were very similar to each other. All three mitogenomes were closed circular molecules ranging from 16,822 to 16,830 bp in length and consisting of 13 PCGs, 22 tRNAs, 2 rRNAs, and a control region (Figure 1). The gene order of the mitogenomes of all three species analyzed were highly conserved (Figure 1), which was also identical to the gene order found in the mitogenome of *Gallus gallus* (Figure 2). For the complete mitogenomes of the three species, one PCG (nad6) and eight tRNAs (*trnQ*, *trnA*, *trnN*, *trnC*, *trnY*, *trnS2*(*UCN*), *trnP*, and *trnE*) were encoded by the L-strand, while all the other genes were encoded by the H-strand. The comparison of the three *Sitta* species showed that the longest overlap (10 bp) was between atp8 and atp6. The longest intergenic spacer (21 bp) was located between *trnV* and *rrnL* in the mitogenome of *S. himalayensis* (Table 2). The mitogenomes of the three *Sitta* species showed a significant bias toward A and T, with the nucleotide composition of A and T ranging from 53.05% to 55.68%. The AT-skew and the GC-skew of the complete mitogenomes of three *Sitta* species were 0.10 to 0.13 and −0.39 to −0.35, respectively (Table 3).

### 3.2. Protein-Coding Genes and Codon Usage

In the three *Sitta* species, the A + T content in PCGs was between 51.97% and 55.17% (Table 3). The start codons and stop codons of the 13 PCGs were mostly the same among the three species; cox1 used GTG as the start codon, while the remaining 12 PCGs initiated strictly with the standard start codon ATG (Table 2). There were six kinds of stop codons (TAA, AGG, AGA, TAG, TA *, and T **) included in the mitogenome of the three *Sitta* species. The PCG nad1 in the mitochondrial genome of *S. nagaensis* contained the incomplete stop codon TA *, while TAN (N represents A and G) occurred in the other two species. Except for nad1, all of the other PCGs used the same stop codon across the three *Sitta* species.

**Figure 3:**
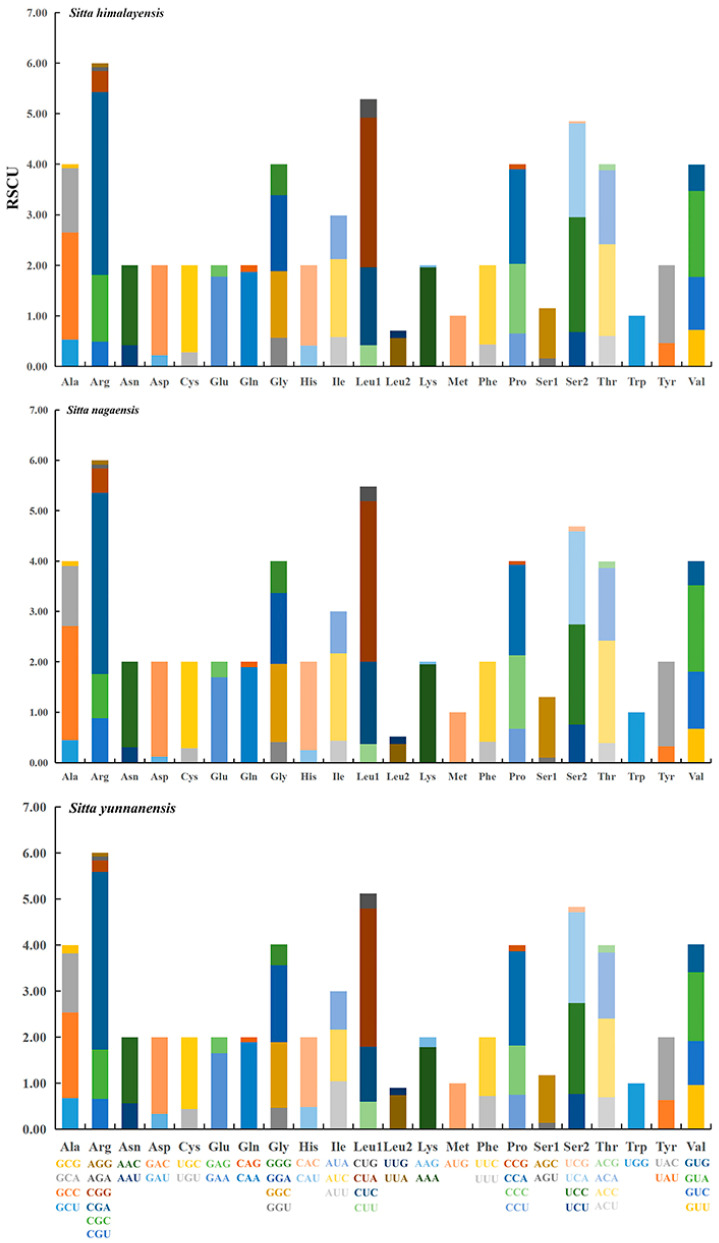
Relative synonymous codon usage (RSCU) for protein-coding genes of the three *Sitta* mitochondrial genomes. Codon families are provided on the x-axis.

The relative synonymous codon usage (RSCU) of 13 PCGs in the three newly sequenced mitogenomes was calculated. As shown in Figure 3, CGA (Arg) and CUA (Leu1) were the most commonly used in all three *Sitta* species. The highest value of RSCU was 3.86 of CGA in *S. yunnanensis* and the lowest value of RSCU was 0.03 of UAG in *S. himalayensis* and *S. nagaensis*. In addition, an analysis of the RSCU values for the 13 PCGs indicated an AT bias. The A + T content in PCGs of *S. yunnanensis* was slightly higher than that of the other two species, and the use of NNA and NNU codons was also more common in *S. yunnanensis*.

### 3.3. tRNA Genes and rRNA Genes

The 22 tRNAs of the three *Sitta* species were typical and included all 20 types of amino acids ranging from 66 to 75 bp in size. The total length of the 22 mitogenome tRNAs of *S. nagaensis* was 1542 bp, which was the same as that of *S. yunnanensis* and only one base lower than that of *S. himalayensis*. The A + T content of the total mitogenome tRNAs of *S. nagaensis* was 58.04%, which was lower than that of *S. himalayensis* (58.26%) and *S. yunnanensis* (58.31%) (Table 3). The tRNAs of the three *Sitta* species were all predicted to fold into typical cloverleaf secondary structures. Furthermore, mismatched base pairs were identified in the stems of 22 different tRNAs, most of which were G-U pairs.

In the mitogenome of the three *Sitta* species, the *16S rRNA* was located between *trnV* and *trnL2*(*UUR*) and ranged from 1575 to 1592 bp in length, while the *12S rRNA* was located between *trnF* and *trnV* and ranged from 977 to 980 bp. The longest *16S rRNA* was found in *S. nagaensis* and the shortest in *S. himalayensis*, while the longest *12S rRNA* was discovered in *S. yunnanensis* and the shortest in *S. himalayensis*. The A + T contents of *16S rRNA* and *12S rRNA* ranged from 55.59% to 56.32% and from 51.17% to 52.25%, respectively (Table 3).

### 3.4. Control Region

**Figure 4:**
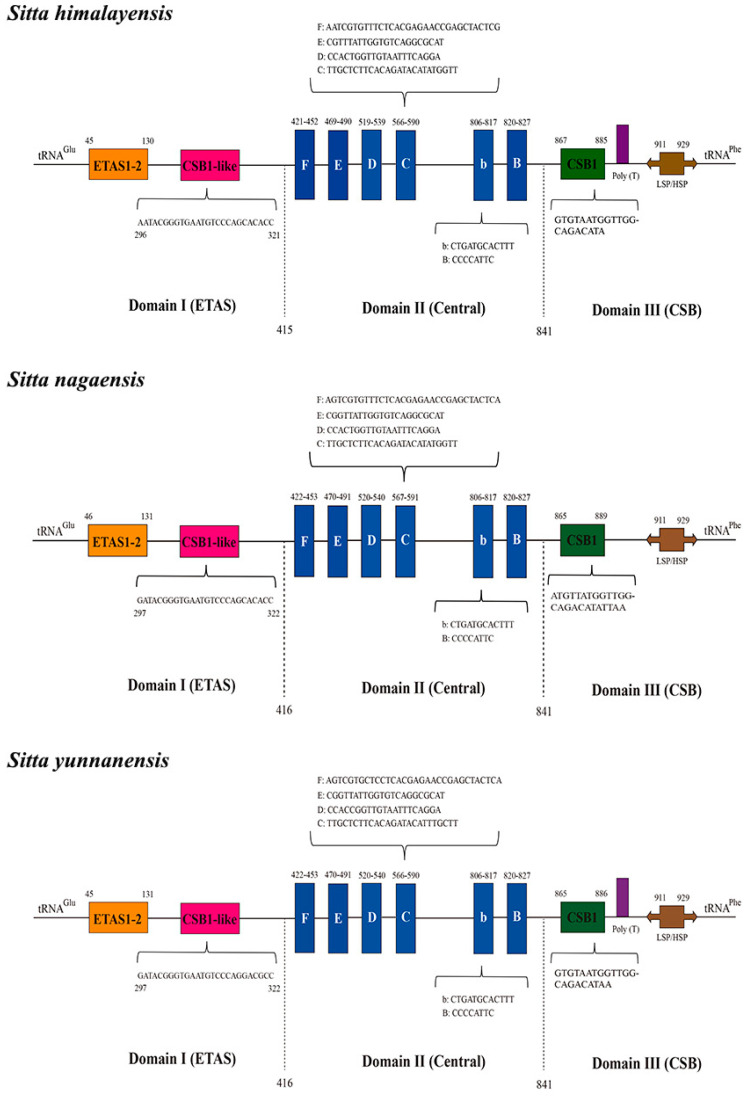
Predicted structural elements in the control region of three *Sitta* species. Extended termination-associated sequences are indicated by orange boxes, and conserved sequence blocks are indicated by blue boxes.

The control region of the three species was located between the *trnE* and *trnF* genes (Figure 1). The size of the control region of *S. yunnanensis* was 975 bp, which was longer than that of *S. himalayensis* (945 bp) and *S. nagaensis* (945 bp). The A + T content of the control region ranged from 53.34% to 55.49% (Table 3). The AT-skew was −0.15 to −0.12, the GC-skew was −0.22, and the A + C content was higher than the T + G content. In this study, we analyzed the control region of three *Sitta* species, and the predicted structures are shown in Figure 4. The entire control region contained three structural domains, namely Domain I, Domain II, and Domain III. Domain II was relatively conservative, while Domain I and Domain III were heterogeneous across species in terms of nucleotide composition and size [42].

Domain I included extended termination-associated sequences such as ETAS1 and ETAS2 and CSB1-like sequences. Domain II was the central conserved domain in the control region and included six conserved sequence blocks (F-box, E-box, D-box, C-box, b-box, and B-box). Domain III included the CSB1 sequence and light/heavy strand promoter (LSP/HSP), which were located at 911–929 bp in the control region (Figure 4).

### 3.5. Phylogenetic Analyses

**Figure 5:**
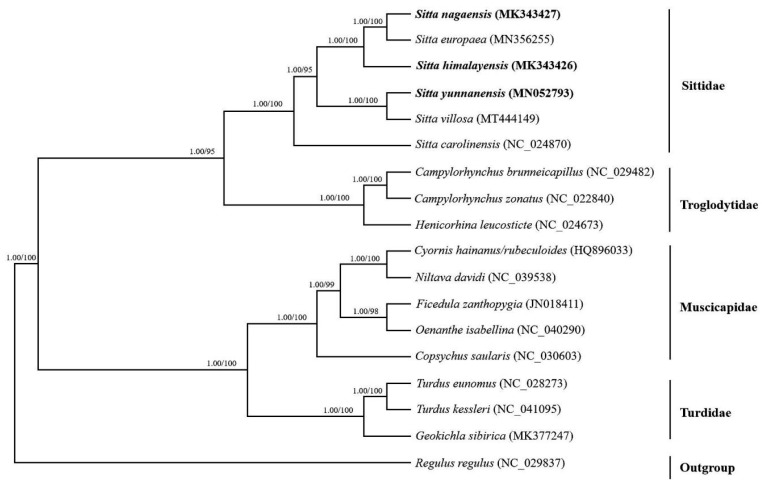
Phylogeny from *Sitta* mitochondrial genome sequences. Bayesian inference (BI) and maximum likelihood (ML) analyses inferred from protein-coding genes supported the same topological structure. Values at nodes are Bayesian posterior probabilities and ML bootstrap values. The tree is rooted with one outgroup (*Regulus regulus*).

All 18 species from Passeriformes (including the three newly sequenced *Sitta* mitogenomes and mitogenomes available from NCBI from three additional *Sitta* species, three Trogloytidae species, five Muscicapidae species, three Turdidae species, and one Regulidae species) were subjected to a phylogenetic analysis based on the concatenated nucleotide sequences of 13 PCGs (Table 1). We used a species of Regulidae as the outgroup based on previously published phylogenies [25,26]. The topologies of phylogenetic analysis in both the BI and ML analyses were identical. The BI posterior probabilities (PPs) and ML bootstrap values (BPs) are labeled in Figure 5.

The present phylogenetic analyses in this study indicated that most species grouped together according to different families. Furthermore, the phylogenetic relationships among four families of the Passeriformes used in this study were: Sittidae and Troglodytidae were clustered into one branch with high nodal support value (PP = 1.00; BP = 95); Muscicapidae and Turdidae were herein corroborated to be sister groups (PP = 1.00; BP = 100); and six species of genus *Sitta* formed a monophyletic group with high support values (PP = 1.00; BP = 100). Within *Sitta,* the phylogenetic topologies revealed that *S. nagaensis*, *S. europaea*, and *S. himalayensis* were closely related and that *S. villosa* and *S. yunnanensis* were closely related. All datasets supported a monophyletic group of *S. carolinensis*, which was placed as a sister to all other species of *Sitta* (PP = 1.00; BP = 100).

## 4. Discussion

### 4.1. Mitogenome Characteristics

The mitochondrial genomes of the three birds sequenced in this study were similar to those of *Sitta* species published in NCBI and ranged from 16,816 to 16,830 bp (*S. villosa*, 16,816 bp; *S. yunnanensis*, 16,830 bp). Variations among mitogenomes in this study were mainly related to the repetition and length of the control regions [43]. The mitogenomes of the three *Sitta* species were similar to those of *Sitta* species published in NCBI: a double-stranded circular molecule composed of 37 typical genes and a control region. The gene order of mitogenomes analyzed was highly conserved, which was also identical to the gene order found in the mitogenome of *Gallus gallus* [44]. Similar to other typical vertebrates, the complete mitogenome of the three *Sitta* species had a high content of A + T and a similar AT/GC-skew [43,45]. The lowest value of the A + T content was 53.05% in *S. nagaensis,* and the highest value of the A+T content was 55.68% in *S. yunnanensis*.

Among the *Sitta* mitogenomes, most PCGs had complete stop codons, while the *cox3* and *nad4* genes had incomplete stop codons (TA *, T **). For the incomplete stop codons, the missing nucleotides may be the result of post-transcriptional polyadenylation [46], which is common in animal mitogenomes and could produce functional stop codons via polycistronic transcription cleavages and polyadenylation mechanisms [46,47]. The patterns of codon usage among the three *Sitta* mitogenomes were nearly the same. The most frequent used codons were NNA and NNU for each amino acid, and the analysis of the RSCU values for the 13 PCGs indicated an AT bias. As for PCGs, the AT bias can be attributed to the frequent use of NNA and NNU codons [48].

All three *Sitta* mitogenomes contained the 22 typical tRNAs, which were all predicted to fold into typical cloverleaf secondary structures, and secondary structures across species were similar. In all published *Sitta* species, the length of *12S rRNA* ranges from 931 bp (*S. europaea*) to 980 bp (*S. yunnanensis*), and the A + T content ranges from 51.17% (*S. himalayensis*) to 52.50% (*S. villosa*). The length of *16S rRNA* ranges from 1575 bp (*S. himalayensis*) to 1597 bp (*S. villosa*), and the A + T content ranges from 55.40% (*S. europaea*) to 56.32% (*S. yunnanensis*). The length and the content of *12S rRNA* and *16S rRNA* genes of the three species involved in this study were within this range. The control region had a rapid evolution rate and was the region with the largest change in the length and sequence of the complete mitochondrial gene [45]. All three species had a control region that showed an obvious AT bias due to the presence of regulatory elements of mitochondrial genome transcription and replication [49].

### 4.2. Phylogenetic Analyses

In this study, a total of 18 species of Passeriformes were selected for phylogenetic analysis to understand the genetic relationship among the genus *Sitta*. The phylogenetic trees generated via BI and ML methods were fully resolved with identical topologies (Figure 5).

The taxonomic status of the Sittidae family is controversial. Cracraft et al. grouped Sittidae and Certhiidae together with Polioptidae and Troglodytidae in the superfamily Certhioidea based on phylogenetic hypotheses [50]. However, the phylogenetic relationship between these four families has not been solved so far within Certhioidea [12]. Treplin et al. placed nuthatches (Sittidae) as a sister of treecreepers (Certhiidae) and the wrens (Troglodytidae) [13]. In this study, we supported the phylogenetic relationships of ((Sittidae + Troglodytidae) + (Muscicapidae + Turdidae)). These phylogenetic relationships were in good accordance with traditional classifications of Sittidae and Troglodytidae [14], whereas recent studies based on morphological data also supported the phylogenetic relationship of ((Polioptidae + Troglodytidae) + (Sittidae + Certhiidae)) [50]. Meanwhile, Muscicapidae and Turdidae as a sister group is well supported [51]. As the mitochondrial genome data of Certhiidae species were not involved in our study, we need to further explore the taxonomic status of nuthatches (Sittidae) by combining the species sequences of multiple families in future studies to confirm the results.

In the phylogenetic relationships within the genus *Sitta*, Päckert et al. estimated divergence times of nuthatches based on the fossil calibration and mean rate estimates for mitochondrial markers and confirmed that *S. carolinensis* was a representative of the first divergent clade among the six *Sitta* species [52]. Meanwhile, *S. nagaensis* and *S. europaea* were found to be the sister to *S. himalayensis*, and *S. villosa* was the sister to *S. yunnanensis*. The same results were obtained in this study, and we also demonstrated the phylogenetic relationships within the genus *Sitta* (*S. carolinensis* + ((*S. villosa* + *S. yunnanensis*) + (*S. himalayensis* + (*S. europaea* + *S. nagaensis*)))). All datasets supported a distant position of *S. carolinensis.* These results were generally identical to those of Pasquet et al. based on molecular markers, biogeography, and life history data [53]. Meanwhile, Pasquet’s work also supported the hypothesis of Asia being the center of diversification for nuthatches (with several independent dispersal events to North America) [53]. Currently, the published mitochondrial genome data of *Sitta* species are very limited, so mitochondrial genomes of more *Sitta* species should be sequenced to better elucidate these phylogenetic relationships.

## 5. Conclusions

In this study, we sequenced three mitochondrial genomes of three *Sitta* species and compared them with related species in terms of the mitochondrial structure and gene rearrangement. The study showed that the mitogenomes of the three species were conserved in genome organization, gene order, and base composition. This study revealed the properties of the mitochondrial genomes of the genus *Sitta* for the first time. Meanwhile, the phylogenetic relationships of the selected species using all PCGs showed that the genus *Sitta* can be divided into three major clades.

## Figures and Tables

**Table 1:** List of 18 species used for the phylogenetic analyses in this study.

Species	GenBank No.	Mitogenome Size (bp)	Total A+T%	References
Sittidae				
*Sitta nagaensis*	MK343427	16,828	53.05	This study
*Sitta* *europaea*	MN356255	16,827	53.19	Unpublished
*Sitta himalayensis*	MK343426	16,822	53.91	This study
*Sitta* *villosa*	MT444149	16,816	55.44	[27]
*Sitta* *yunnanensis*	MN052793	16,830	55.68	This study
*Sitta* *carolinensis*	NC_024870	16,826	54.78	[14]
Troglodytidae				
*Campylorhynchus zonatus*	NC_022840	16,780	50.57	[28]
*Campylorhynchus brunneicapillus*	NC_029482	16,786	50.34	Unpublished
*Henicorhina leucosticta*	NC_024673	16,727	52.88	[29]
Muscicapidae				
*Oenanthe isabellina*	NC_040290	16,812	52.66	[30]
*Ficedula zanthopygia*	JN018411	16,794	53.16	Unpublished
*Niltava davidi*	NC_039538	16,770	54.16	Unpublished
*Cyornis hainanus/rubeculoides*	HQ896033	16,802	53.01	Unpublished
*Copsychus saularis*	NC_030603	16,827	52.74	[31]
Turdidae				
*Turdus kessleri*	NC_041095	16,754	52.71	[32]
*Turdus eunomus*	NC_028273	16,737	52.69	Unpublished
*Geokichla sibirica*	MK377247	16,766	52.28	[33]
Regulidae				
*Regulus regulus*	NC_029837	16,847	55.52	Unpublished

**Table 2:** Annotation of the complete mitogenome of the three *Sitta* species in this study.

Gene	Start Codon	Stop Codon	Anti-Codon	Strand	Intergenic Nucleotides (IGN)
*trnF*			GAA	H	−1
*rrnS*				H	0(a)/−1(b,c)
*trnV*			TAC	H	21(a)/2(b,c)
*rrnL*				H	2(a)/1(b)/7(c)
*trnL2*(*UUR*)			TAA	H	11(a,c)/12(b)
nad1	ATG	TAA/TA(A)/TAG		H	7(a,c)/10(b)
*trnI*			GAT	H	6
*trnQ*			TTG	L	4(a)/3(b)/2(c)
*trnM*			CAT	H	1(a,b)/0(c)
nad2	ATG	TAA		H	1
*trnW*			TCA	H	1
*trnA*			TGC	L	10
*trnN*			GTT	L	2(a,c)/11(b)
*trnC*			GCA	L	−1
*trnY*			GTA	L	1
cox1	GTG	AGG		H	−9
*trnS2*(*UCN*)			TGA	L	4
*trnD*			GTC	H	10(a)/11(b,c)
cox2	ATG	TAA		H	1
*trnK*			TTT	H	1
atp8	ATG	TAA		H	−10
atp6	ATG	TAA		H	9(a,b)/11(c)
cox3	ATG	TA(A)		H	−1
*trnG*			TCC	H	0
nad3	ATG	TAA		H	−1
*trnR*			TCG	H	1
nad4L	ATG	TAA		H	−7
nad4	ATG	T(AA)		H	0
*trnH*			GTG	H	0
*trnS1*(*AGN*)			GCT	H	−1
*trnL1*(*CUN*)			TAG	H	1(a)/0(b,c)
nad5	ATG	AGA		H	11
Cytb	ATG	TAA		H	6(a)/3(b,c)
*trnT*			TGT	H	7(a)/8(b,c)
*trnP*			TGG	L	14(a,b)/6(c)
nad6	ATG	TAG		L	0
*trnE*			TTC	L	4(a)/5(b,c)
CR				H	279(a)/282(b)/266(c)

Note: H and L refer to the heavy and light strands; CR = control region. ‘/’ indicates type of intergenic nucleotides in *Sitta* species: *S. himalayensis* (a); *S. nagaensis* (b); *S. yunnanensis* (c).

**Table 3:** Nucleotide composition of the mitochondrial genome of the three *Sitta* species in this study.

Species	Whole Genome			PCGs			tRNAs			*16S rRNA*			*12S rRNA*			Control Region		
	A+T (%)	AT Skew	GC Skew	A+T (%)	AT Skew	GC Skew	A+T (%)	AT Skew	GC Skew	A+T (%)	AT Skew	GC Skew	A+T (%)	AT Skew	GC Skew	A+T (%)	AT Skew	GC Skew
*S. himalayensis*	53.91	0.13	−0.38	53.09	0.07	−0.41	58.26	0.03	0.02	55.62	0.24	−0.12	51.17	0.18	−0.11	54.60	−0.15	−0.22
*S. nagaensis*	53.05	0.13	−0.39	51.97	0.09	−0.42	58.04	0.04	0.01	55.59	0.24	−0.11	51.44	0.18	−0.11	53.34	−0.12	−0.22
*S. yunnanensis*	55.68	0.10	−0.35	55.17	0.04	−0.38	58.31	0.04	0.01	56.32	0.23	−0.10	52.25	0.18	−0.11	55.49	−0.15	−0.22

## Data Availability

Not applicable.

## References

[1] Wolstenholme D.R. (1992). Animal mitochondrial DNA: Structure and evolution. Int. Rev. Cytol..

[2] Boore J.L. (1999). Animal mitochondrial genomes. Nucleic Acids Res..

[3] Taanman J.W. (1999). The mitochondrial genome: Structure, transcription, translation and replication. Biochim. Biophys. Acta.

[4] Li Q.W., Li S., Tian C.Y., Wang Y.J., Guo Y.M. (2002). Molecular evolution and variability in mitochondrial DNA in ten species of Passeriforme. Acta Zool. Sin..

[5] Quinn T.W. (1997). Molecular Evolution of the Mitochondrial Genome—Chapter 1. Avian Molecular Evolution and Systematics.

[6] Kan X.Z., Li X.F., Lei Z.P., Wang M., Chen L., Gao H., Yang Z.Y. (2010). Complete mitochondrial genome of Cabot’s tragopan, *Tragopan caboti* (Galliformes: Phasianidae). Genet. Mol. Res..

[7] Zhou C., Hao Y.Q., Ma J.N., Zhang W.B., Chen Y.Z., Chen B.P., Zhang X.Y., Yue B.S. (2017). The first complete mitogenome of *Picumnus innominatus* (Aves, Piciformes, Picidae) and phylogenetic inference within the Picidae. Biochem. Syst. Ecol..

[8] Sangster G., Luksenburg J.A. (2021). Sharp increase of problematic mitogenomes of birds: Causes, consequences, and remedies. Genome Biol. Evol..

[9] HBW-BirdLife (2022). Handbook of the Birds of the World and Birdlife, Version 2022-7. http://datazone.birdlife.org.

[10] Ericson P.G.P., Johansson U.S. (2003). Phylogeny of Passerida (Aves: Passeriformes) based on nuclear and mitochondrial sequence data. Mol. Phylogenet. Evol..

[11] Johansson U.S., Fjeldså J., Bowie R.C. (2008). Phylogenetic relationships within Passerida (Aves: Passeriformes): A review and a new molecular phylogeny based on three nuclear intron markers. Mol. Phylogenet. Evol..

[12] Zhao M., Alström P., Olsson U., Qu Y.H., Lei F.M. (2016). Phylogenetic position of the Wallcreeper *Tichodroma muraria*. J. Ornithol..

[13] Treplin S., Siegert R., Bleidorn C., Thompson H.S., Fotso R., Tiedemann R. (2008). Molecular phylogeny of songbirds (Aves: Passeriformes) and the relative utility of common nuclear marker loci. Cladistics.

[14] Barker F.K. (2014). Mitogenomic data resolve basal relationships among passeriform and passeridan birds. Mol. Phylogenet. Evol..

[15] Chen Z.T., Du Y.Z. (2017). First mitochondrial genome from Nemouridae (Plecoptera) reveals novel features of the elongated control region and phylogenetic implications. Int. J. Mol. Sci..

[16] Liu G., Zhou L.Z., Li B., Zhang L.L. (2014). The complete mitochondrial genome of *Aix galericulata* and *Tadorna ferruginea*: Bearings on their phylogenetic position in the Anseriformes. PLoS One.

[17] Zhang Z.C., Cheng Q.Q., Ge Y.S. (2019). The complete mitochondrial genome of *Rhynchocypris oxycephalus* (Teleostei: Cyprinidae) and its phylogenetic implications. Ecol. Evol..

[18] Lu X.T., Gong L., Zhang Y., Chen J., Liu L.Q., Jiang L.H., Lü Z.M., Liu B.J., Tong G.X., Wei X.X. (2020). The complete mitochondrial genome of *Calappa bilineata*: The first representative from the family Calappidae and its phylogenetic position within Brachyura. Genomics.

[19] Lohse M., Drechsel O., Bock R. (2007). OrganellarGenomeDRAW (OGDRAW): A tool for the easy generation of high-quality custom graphical maps of plastid and mitochondrial genomes. Curr. Genet..

[20] Laslett D., Canback B. (2008). ARWEN: A program to detect tRNA genes in metazoan mitochondrial nucleotide sequences. Bioinformatics.

[21] Lowe T.M., Chan P.P. (2016). tRNAscan-SE On-line: Integrating search and context for analysis of transfer RNA genes. Nucleic Acids Res..

[22] Kumar S., Stecher G., Tamura K. (2016). MEGA7: Molecular evolutionary genetics analysis version 7.0 for bigger datasets. Mol. Biol. Evol..

[23] Perna N.T., Kocher T.D. (1995). Patterns of nucleotide composition at fourfold degenerate sites of animal mitochondrial genomes. J. Mol. Evol..

[24] Gary B. (1999). Tandem repeats finder: A program to analyze DNA sequences. Nucleic Acids Res..

[25] Oliveros C.H., Field D.J., Ksepka D.T., Barker F.K., Aleixo A., Andersen M.J., Alström P., Benz B.W., Braun E.L., Braun M.J. (2019). Earth history and the passerine superradiation. Proc. Natl. Acad. Sci. USA.

[26] Stervander M., Fjeldså J., Christidis L., Ericson P.G.P., Ohlson J.I., Alström P., Fjeldså J., Christidis L., Ericson P.G.P. (2020). An updated chronology of passerine birds. The Largest Avian Radiation.

[27] Zhang Z.R., Mi S.H., Guo Q.X., Zhang Z., Yan P.F., Liu Z.S., Teng L.W. (2020). The complete mitochondrial genome of the *Sitta villosa* (Passeriformes: Sittidae) from China. Mitochondrial DNA Part B.

[28] Barker F.K., Oyler-McCance S., Tomback D.F. (2013). Blood from a turnip: Tissue origin of low-coverage shotgun sequencing libraries affects recovery of mitogenome sequences. Mitochondrial DNA.

[29] Aguilar C., Léon L.F.D., Loaiza J.R., McMillan W.O., Miller M.J. (2016). Extreme sequence divergence between mitochondrial genomes of two subspecies of White-breasted Wood-wren (*Henicorhina leucosticte*, Cabanis, 1847) from western and central Panamá. Mitochondrial DNA.

[30] Li S.B., Luo A., Li G.P., Li W. (2016). Complete mitochondrial genome of the isabelline wheatear *Oenanthe isabellina* (Passeriformes, Muscicapidae). Mitochondrial DNA Part B.

[31] Peng L.F., Yang D.C., Lu C.H. (2016). Complete mitochondrial genome of oriental magpie-robin *Copsychus saularis* (Aves: Muscicapidae). Mitochondrial DNA Part B.

[32] Song S., Qin J.H., Luo J.J., Li D.H., Jiang B., Chang C. (2018). Analysis of complete mitochondrial genome sequence of Kessleri thrush, *Turdus kessleri* (Passeriformes, Turdidae). Mitochondrial DNA Part B.

[33] Sun C.H., Liu B., Lu C.H. (2019). Complete mitochondrial genome of the Siberian thrush, *Geokichla sibirica sibirica* (Aves, Turdidae). Mitochondrial DNA Part B.

[34] Larkin M.A., Blackshields G., Brown N.P., Chenna R., McGettigan P.A., McWilliam H., Valentin F., Wallace I.M., Wilm A., Lopez R. (2007). ClustalW and ClustalX version 2.0. Bioinformatics.

[35] Zhou X.P., Lin Q.X., Fang W.Z., Chen X.L. (2014). The complete mitochondrial genomes of sixteen ardeid birds revealing the evolutionary process of the gene rearrangements. BMC Genom..

[36] Santorum J.M., Darriba D., Taboada G.L., Posada D. (2014). Jmodeltest.org: Selection of nucleotide substitution models on the cloud. Bioinformatics.

[37] Ronquist F., Huelsenbeck J.P. (2003). MrBayes 3: Bayesian phylogenetic inference under mixed models. Bioinformatics.

[38] Ronquist F., Teslenko M., Mark P.V.D., Ayres D.L., Darling A., Höhna S., Larget B., Liu L., Suchard M.A., Huelsenbeck J.P. (2012). MrBayes 3.2: Efficient Bayesian phylogenetic inference and model choice across a large model space. Syst. Biol..

[39] Guindon S., Gascuel O. (2003). A simple, fast, and accurate algorithm to estimate large phylogenies by maximum likelihood. Syst. Biol..

[40] Huelsenbeck J.P., Ronquist F., Nielsen R., Bollback J.P. (2001). Bayesian inference of phylogeny and its impact on evolutionary biology. Science.

[41] Rambaut A., Drummond A.J. Figtree Version 1.4.0. http://tree.bio.ed.ac.uk/software/figtree.

[42] Huang Z.H., Liao X.J. (2011). Structure of the mitochondrial DNA control region and genetic variation of *Chrysolophus pictus*. Life Sci. Res..

[43] Xiao B., Ma F., Sun Y., Li Q.W. (2006). Comparative analysis of complete mitochondrial DNA control region of four species of Strigiformes. Acta Genet. Sin..

[44] Dejardins P., Morais R. (1990). Sequence and gene organization of the chicken mitochondrial genome: A novel gene order in higher vertebrates. J. Mol. Biol..

[45] Shadel G.S., Clayton D.A. (1997). Mitochondrial DNA maintenance in vertebrates. Annu. Rev. Biochem..

[46] Ojala D., Montoya J., Attardi G. (1981). tRNA punctuation model of RNA processing in human mitochondria. Nature.

[47] Boore J.L. (2001). Complete mitochondrial genome sequence of the polychaete annelid *Platynereis dumerilii*. Mol. Biol. Evol..

[48] Li W.J., Wang Z.Q., Che Y.L. (2017). The complete mitogenome of the Wood-Feeding Cockroach *Cryptocercus meridianus* (Blattodea: Cryptocercidae) and its phylogenetic relationship among cockroach families. Int. J. Mol. Sci..

[49] Zhu X.Y., Xin Z.Z., Wang Y., Zhang H.B., Zhang D.Z., Wang Z.F., Zhou C.L., Tang B.P., Liu Q.N. (2017). The complete mitochondrial genome of *Clostera anachoreta* (Lepidoptera: Notodontidae) and phylogenetic implications for Noctuoidea species. Genomics.

[50] Cracraft J., Barker F.K., Braun M., Harshman J., Dyke G.J., Feinstein J., Stanley S., Cibois A., Schikler P., Beresford P., Cracraft J., Donoghue M. (2004). Phylogenetic relationships among modern birds (Neornithes): Towards an avian tree of life. Assembling the Tree of Life.

[51] Tobias J.A., Sheard C., Pigot A.L., Devenish A.J.M., Yang J.Y., Sayol F., Neate-Clegg M.H.C., Alioravainen N., Weeks T.L., Barber R.A. (2022). A VONET: Morphological, ecological and geographical data for all birds. Ecol. Lett..

[52] Päckert M., Bader-Blukott M., Künzelmann B., Sun Y.H., Hsu Y.C., Kehlmaier C., Albrecht F., Illera J.C., Martens J. (2020). A revised phylogeny of nuthatches (Aves, Passeriformes, *Sitta*) reveals insight in intra- and interspecific diversification patterns in the Palearctic. Vertebr. Zool..

[53] Pasquet E., Barker F.K., Martens J., Tillier A., Cruaud C., Cibois A. (2014). Evolution within the nuthatches (Sittidae: Aves, Passeriformes): Molecular phylogeny, biogeography, and ecological perspectives. J. Ornithol..

